# Painful detour of subcutaneous implantable cardioverter-defibrillator lead

**DOI:** 10.1016/j.hrcr.2022.02.009

**Published:** 2022-02-24

**Authors:** Jasmine Bisson, Marc Dubuc, Stéphanie Tan, Blandine Mondésert

**Affiliations:** ∗ Division of Cardiology, Medicine Department, Montreal Heart Institute, University of Montreal, Montreal, Canada; † Division of Electrophysiology, Medicine Department, Montreal Heart Institute, University of Montreal, Montreal, Canada; ‡ Division of Radiology, Medicine Department, Montreal Heart Institute, University of Montreal, Montreal, Canada

**Keywords:** S-ICD, Complication, Lead, Electrode, Intercostal, Intrathoracic, Subcutaneous, Revision

**Key Teaching Points**

• Intercostal trajectory of subcutaneous implantable cardioverter-defibrillator (S-ICD) lead is a rare but now acknowledged complication of S-ICD placement.
• Clinical presentation can include refractory pain (that might be delayed) and evidence of inflammation of the surrounding tissues on positron emission tomography scan.
• Extraction and revision of S-ICD placement can be safely performed under careful monitoring.
• Computed tomography scan is the preferred modality for diagnosis of lead mispositioning, but it warrants specific attention to lead trajectory.

## Introduction

Utilization of the subcutaneous implantable cardioverter-defibrillator (S-ICD) has increased over the past decade, as an extravascular alternative to the transvenous ICD. With this growing experience, we are facing new complications. This case report aims to outline a unique complication of an intrathoracic S-ICD lead positioning.

## Case report

We report the case of a 34-year-old woman who underwent an evaluation for ICD placement after sudden cardiac death. During a hospitalization for an acute systemic lupus erythematosus flare-up, she experienced 2 consecutive episodes of polymorphic ventricular tachycardia requiring external cardiac defibrillation to restore sinus rhythm.

Subsequent cardiac magnetic resonance imaging revealed normal left and right ventricular functions, with extensive (10%–15% of left ventricle) late gadolinium enhancement compatible with scar from prior lupus myocarditis. In addition, a small ostium secundum atrial septal defect was described on transthoracic echocardiography. In light of all this, an S-ICD in secondary prevention was chosen to prevent both infection in an immunosuppressed patient and stroke in a patient with an atrial septal defect. Moreover, she was at low risk of pacing requirement.

**Figure 1:**
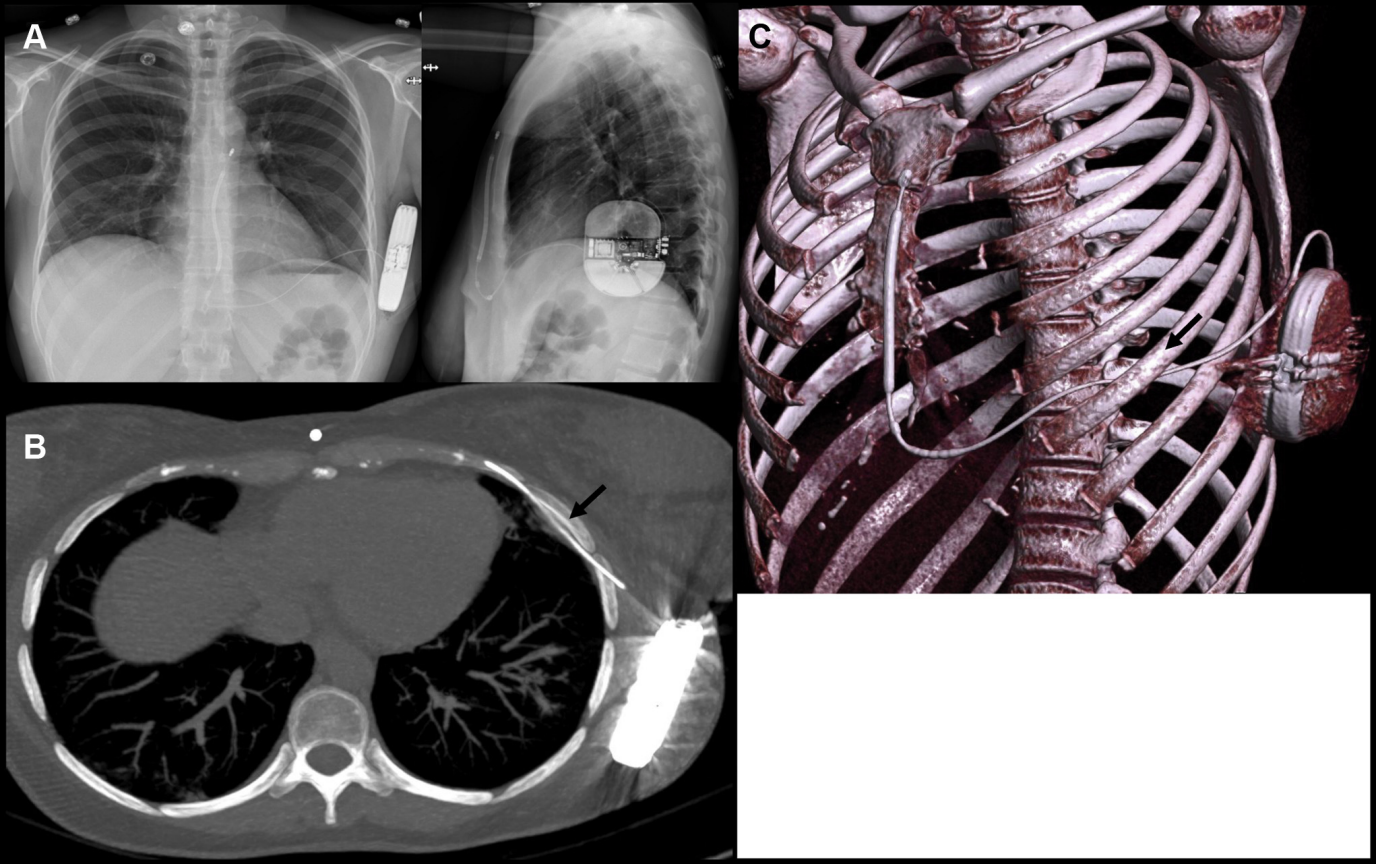
**A:** Chest radiograph after initial subcutaneous implantable cardioverter-defibrillator (S-ICD) implantation. **B, C:** Chest computed tomography with maximum-intensity projection (B) and volume rendering (C) showing intrathoracic trajectory of S-ICD lead (*black arrows*).

She eventually underwent implantation of an S-ICD (EMBLEM MRI S-ICD; Boston Scientific, Marlboro, MA) with a standard intermuscular 2-incision approach in August 2020. Defibrillation testing was performed with a ventricular tachycardia well recognized and treated with an 80 J shock. Shock impedance was 64 ohms. The procedure, which was done under local anesthesia and conscious sedation, was well tolerated. The patient was discharged the day after, without complaint. Radiography confirmed adequate positioning of the cardiac device with a PRAETORIAN score of 30, indicating low risk of conversion failure^1^ (Figure 1A).

In January 2021, the patient first mentioned pain around the generator. It then increased gradually, to the point where in March she could no longer tolerate wearing a bra. Neurodystrophic reaction was hypothesized and a regimen of pregabalin and acetaminophen was prescribed, with only mild improvement of symptoms. To rule out an occult infection process, a fluorodeoxyglucose–positron emission tomography (PET) scan was performed and showed hypermetabolism around the generator and the first 5 centimeters of the lead. More interestingly, the S-ICD lead was passing under the sixth rib and in the subpleural space before re-emerging in the subcutaneous tissues. This latter finding was confirmed with high-resolution computed tomography (CT) scan (Figure 1B and 1C). An initial conservative medical management was favored, with antibiotics and pain killers and with a close follow-up scheduled.

One month later, unchanged PET scan findings and refractory clinical symptoms despite antibiotics led to an inflammatory hypothesis, and the patient was scheduled for S-ICD revision.

**Figure 2:**
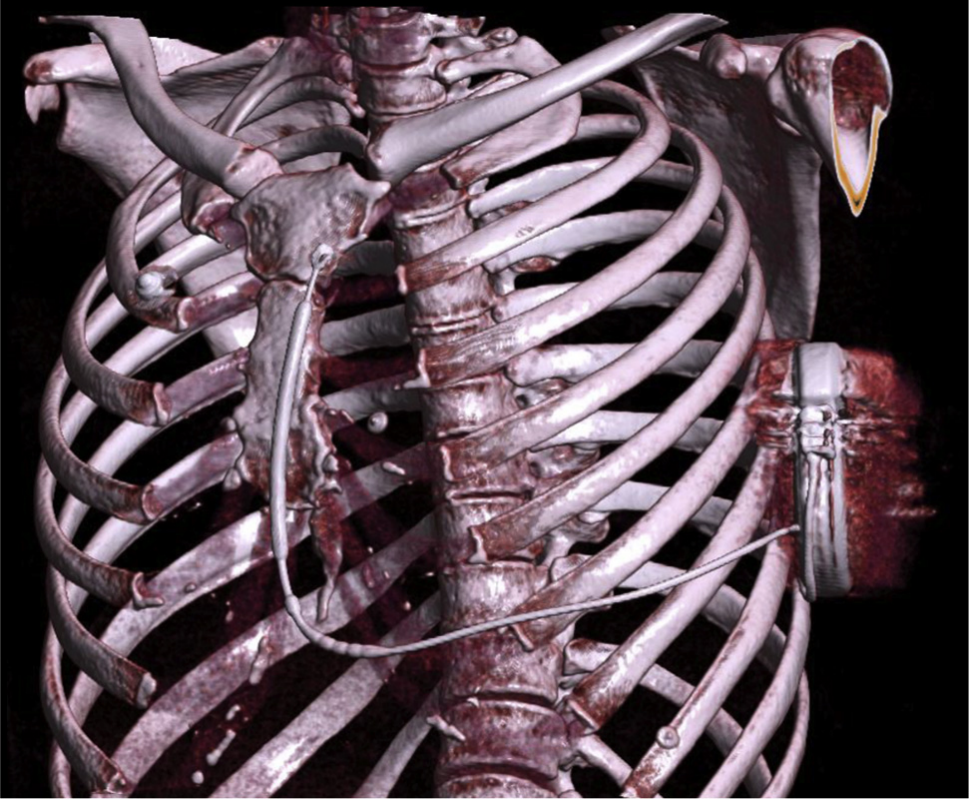
Chest computed tomography with volume rendering showing the repositioned subcutaneous implantable cardioverter-defibrillator lead in correct position within the subcutaneous tissue.

On April 2021, the S-ICD system was completely extracted under local anesthesia and conscious sedation. The lateral pocket was reopened without any sign of infection. The lead was removed by a gentle traction through the parasternal incision. There was no pneumothorax, and a new lead was carefully reimplanted in the subcutaneous tissues, using the same parasternal incision. Defibrillation test was once again performed and was successful. The patient received her discharge the same day. Shortly after the S-ICD revision, the localized pain completely resolved. At 1-month follow-up, a CT-scanner was performed to document the final subcutaneous positioning of the S-ICD system (Figure 2). The patient remains free of symptoms on follow-up 9 months later.

## Discussion

To our knowledge, this is the first report of an inadvertent intercostal trajectory of the horizontal portion of the subcutaneous lead. In the PRAETORIAN study, only 2 out of 426 patients assigned to S-ICD required lead repositioning (compared to 7 out of 423 in the transvenous ICD group), although the reasons for repositioning were not explicitly stated.^2^ In the pooled results of the IDE and EFORTLESS trials, 12 patients ended with suboptimal lead positioning, without specific mention of intrathoracic trajectory of the lead in either.^3^ One Spanish team did report the case of the parasternal portion of the lead passing through an intercostal space to a retrosternal position, with its tip plunging toward the pulmonary artery, in a patient with significant chest malformation (severe pectus excavatum with many prior surgical revisions). In that case, the lead was repositioned the next day with simple traction as well.^4^

In our case, the mispositioning of the lead was not initially recognized, as all usual indicators of successful implantation were present. The later clinical presentation was mainly driven by the pain localized around the generator, as radiated pain coming from the intercostal inflammation secondary to the uncommon lead mispositioning. The friction of the lead on the rib and surrounding intercostal muscles with each respiratory movement could be involved in developing and maintaining the inflammatory response. It is, however, surprising that pain only arose 5 months after the procedure. In terms of implantation techniques, it has always been emphasized to put the lead deep on the sternum bone to decrease the energy needed for defibrillation. However, in this case, particular attention to stay in subcutaneous tissues remains important. Tunneling the lead from the sternal incision toward the lateral pocket (and not the opposite way) seems to be the safest approach to avoid that kind of complication and the wrong route of the lead.

The low-dose CT scan, performed in conjunction with the PET scan for anatomic correlation, has been the exam that provided the diagnosis of the unfortunate lead trajectory. Interestingly, the patient had a previous CT scan done at another center for a different condition, but the lead mispositioning was not reported at the time. The S-ICD being a relatively new device, some radiologists could be unfamiliar with normal localization and trajectory of the subcutaneous lead.

Finally, complete extraction and repositioning of the lead system resolved the symptoms entirely. Even though the S-ICD had been placed 8 months earlier, the lead was successfully extracted in 1 piece with simple traction from the parasternal incision, without complication. The lead has been sent for analysis to Boston Scientific facilities, and no abnormalities have been reported, especially on the intercostal part. Theoretically, the lead could have been simply repositioned without extracting the parasternal part.

In conclusion, unfortunate intercostal trajectory of the S-ICD lead is a rare complication of S-ICD implantation of which electrophysiologists and radiologists should be made aware. It may manifest as localized neurologic pain and can be diagnosed with a chest CT. In our case, surgical revision and lead repositioning was successful in resolving all symptoms.
